# Macromolecular-clustered facial amphiphilic antimicrobials

**DOI:** 10.1038/s41467-018-07651-7

**Published:** 2018-12-07

**Authors:** Md Anisur Rahman, Marpe Bam, Edgar Luat, Moumita Sharmin Jui, Mitra S. Ganewatta, Tinom Shokfai, Mitzi Nagarkatti, Alan W. Decho, Chuanbing Tang

**Affiliations:** 1 Department of Chemistry and Biochemistry, Columbia, SC 29208 United States; 2 Department of Pathology, Microbiology and Immunology, University of South Carolina, School of Medicine, Columbia, SC 29209 United States; 3 Department of Environmental Health Sciences, University of South Carolina, Columbia, SC 29208 United States

## Abstract

Bacterial infections and antibiotic resistance, particularly by Gram-negative pathogens, have become a global healthcare crisis. We report the design of a class of cationic antimicrobial polymers that cluster local facial amphiphilicity from repeating units to enhance interactions with bacterial membranes without requiring a globally conformational arrangement associated with highly unfavorable entropic loss. This concept of macromolecular architectures is demonstrated with a series of multicyclic natural product-based cationic polymers. We have shown that cholic acid derivatives with three charged head groups are more potent and selective than lithocholic and deoxycholic counterparts, particularly against Gram-negative bacteria. This is ascribed to the formation of true facial amphiphilicity with hydrophilic ion groups oriented on one face and hydrophobic multicyclic hydrocarbon structures on the opposite face. Such local facial amphiphilicity is clustered via a flexible macromolecular backbone in a concerted way when in contact with bacterial membranes.

## Abstract (web summary)

Bacterial infections and antibiotic resistance are an increasing concern for the world. Here the authors report a class of cationic antimicrobial polymers that cluster local facial amphiphilicity from repeating units to enhance interactions with bacterial membranes without requiring a high unfavorable entropic loss.

## Introduction

Antimicrobial resistance is an ever-increasing threat to public health, and is projected to be accountable for more deaths than cancer and AIDS combined by 2050^1,2^. The effective treatments for bacterial infections are becoming radically diminished as bacteria develop resistance against most available antibiotics^3^. Among these multidrug-resistant (MDR) pathogens, Gram-negative bacteria pose more perilous threats to human life^4^. Most infections caused by Gram-negative MDR bacteria are essentially untreatable, and may lead to severe illness or even death^4,5^. Despite this fact, the development of new antimicrobial therapies has been primarily focused on Gram-positive bacteria^6,7^. The presence of dual membranes in Gram-negative bacteria acts as an impermeable barrier to most antibiotics. As a result, there arises an urgent need for new-generation antimicrobials with potent therapeutic activity, novel modes of action, and without driving the current increase of antimicrobial resistance, especially to combat the growing epidemic of infections caused by MDR pathogens.

**Fig. 1:**
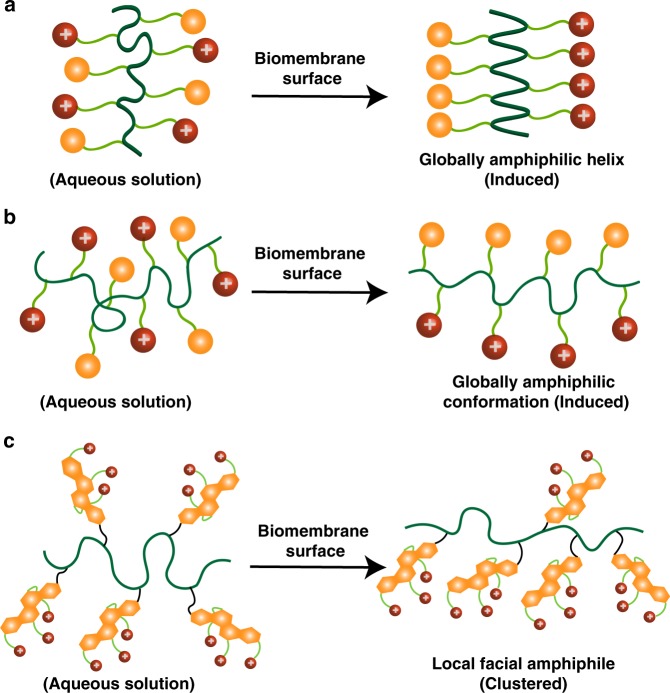
Modes of action adopted upon approaching to a biomembrane surface. **a** Host-defense peptides adopting a globally amphiphilic helical conformation^36^; **b** Synthetic antimicrobial polymers adopting a globally amphiphilic conformation; and **c** A flexible macromolecular chain clustering intrinsic local facial amphiphiles (this work). Red color: cationic/hydrophilic groups, yellow color: hydrophobic groups

Natural antimicrobial peptides (AMPs) are amphiphilic, combining cationic charges and hydrophobic components, and able to electrostatically bind to anionic bacterial membranes or other anionic targets^8–13^. It is well known that in many cases, AMPs form an α-helix structure with positive charges arrayed on one side and lipophilic groups aligned along the other side in contact with bacterial membranes (Fig. 1a)^14–16^. The common structural features of these AMPs with a global segregation of cationic and lipophilic side chains are also referred to as facial amphiphilicity (i.e. separate hydrophilic and hydrophobic faces)^16,17^. Facial amphiphilicity allows AMPs to efficiently insert into bacterial membranes via the barrel-stave pore, toroidal pore, disordered toroidal pore, and/or carpet mechanisms, leading to cytoplasmic leakage, membrane depolarization, lysis, and cell death^18,19^. Over the last two decades, natural AMP-mimicking peptide derivatives such as β-peptides and peptoids have been developed with potent antimicrobial activity^20–23^. However, the clinical applications of AMPs are very limited due to their low bioavailability, low stability, high manufacturing cost, as well as in many cases nonspecific toxicity to mammalian cells^7,13,19,24^. To address these issues, synthetic polymers with cationic charges, which mimic natural AMPs and selectively attack negative bacterial cell membranes over zwitterionic mammalian cell membranes, have been studied widely as a promising solution to combat bacteria. These polymers offer a broad spectrum of antimicrobial activity, a membrane disruption mechanism as well as a low propensity for developing resistance^13,18,25,26^. In addition, cationic charge-containing polymers can be obtained in large quantities at much lower cost. Many antimicrobial polymers are highly effective in killing traditional strains. We have developed several antimicrobial macromolecules utilizing bulky hydrophobic structures containing natural resin acids and antibiotic-metal bioconjugates that exhibit excellent activities against bacteria, particularly against Gram-positive bacteria such as methicillin-resistant *Staphylococcus aureus* (MRSA), while simultaneously exhibiting low hemolysis against red blood cells and minimal in vitro and in vivo cytotoxicity^27–35^.

However, most antimicrobial polymers with AMP-mimicking designs are based on the adoption of a conformation that is globally amphiphilic, which requires control on the sequence of hydrophobic and hydrophilic subunits. Gellman and coworkers stated that the facial amphiphilicity could be achieved from random copolymerization of hydrophobic and hydrophilic monomers that did not require control of subunit sequences^16,36,37^. Their copolymers contained both cationic and lipophilic groups as well as sufficiently flexible backbones that could form a globally amphiphilic, but conformationally irregular helical structure induced by negatively charged bacterial membranes (Fig. 1b). DeGrado, Kuroda and coworkers also synthesized methacrylate-based copolymers consisting of flexible backbones and amphiphilic compositions with low toxicity and good antimicrobial activity^38,39^. Tew et al. synthesized amphiphilic cationic polymers that also exhibited good antimicrobial activity, where they used amphiphilic monomers (i.e., containing both a hydrophilic ammonium and a hydrophobic norbornene on the same polymerizable unit)^40,41^.

However, most of these approaches rely on uncontrolled polymeric self-aggregation to achieve global facial amphiphilicity, which is difficult to manipulate. From the perspective of free energy change upon the contact with bacterial cell membranes, the fact of adopting a facial amphiphilic conformation without the helical structures from random coil structures of synthetic polymers would suffer a very high entropic penalty from a whole macromolecule (Fig. 1b).

In fact, most antimicrobial polymers do not comprise truly facial amphiphilicity and suffer poor selectivity and high cytotoxicity against mammalian cells and are also ineffective against MDR Gram-negative bacteria. We hypothesized that a flexible macromolecule carrying intrinsic facial amphiphilic units with a large cross-sectional area would offer a novel type of antimicrobial polymer, in which each local unit could exert an insertable handle upon contact with bacterial membranes. The polymeric backbone not only avoids adopting a highly energetic, global amphiphilicity, but also assembles the intrinsic local facial amphiphilic structures on cell membranes. The macromolecular structures would significantly increase the density of local facial amphiphilicity and thus enhance the overall interactions with bacterial cells. To test this hypothesis, we chose multicyclic natural products, e.g. steroid acids or terpenoids, as a functionalized building block to possess local facial amphiphilicity.

**Fig. 2:**
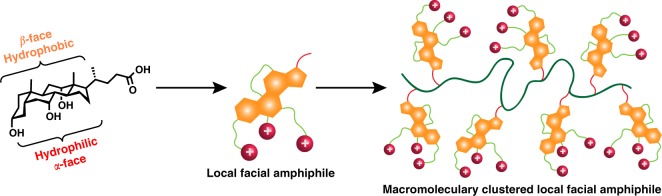
Design principle of cationic polymers with an intrinsic facial amphiphilic structure at repeat units. The key building block should have a multicyclic structure with the possibility for derivatization to possess one face hydrophilic and the other face hydrophobic. Cholic acid is illustrated as an example here

Bile acids are cholesterol-derived amphiphilic steroid acids produced in mammals and other vertebrates. They have been utilized in many areas including drug delivery, sensors, polymeric gels, antimicrobials, and other biological applications^35,42–44^. There are four different derivatives of bile acids, which vary by the number of hydroxyl groups, such as cholic acid (CA), deoxycholic acid (DCA), chenodeoxycholic acid, and lithocholic acid (LCA) (Supplementary Figure 1a). Hydroxyl groups of bile acid molecules are positioned in the concave *α*-face while the multicyclic hydrocarbon structure is constituted as the convex *β*-face, thereby providing the potential to achieve true facial amphiphilicity (Fig. 2). The steroidal nucleus with four fused rings provides a hydrophobic core with a significantly larger cross-sectional area compared to linear alkyl chains. The facial amphiphilicity, biocompatibility, and hydrophobicity of bile acid derivatives are considered highly favorable for interactions with bacterial cell membranes.

Herein we report the synthesis of cationic bile acid-based polymers that possess intrinsic local facial amphiphilicity clustered together via a flexible macromolecular chain (Fig. 2). The presence of hydroxyl groups from the *α*-face allows the installation of cationic quaternary ammonium charges (QAC) as hydrophilic components. The carboxylic acid at the edge of this particular structure offers chemical functionalization for the attachment as a pendant monomeric unit integrated into a flexible macromolecular skeleton. Three different bile acid derivatives, lithocholic, deoxycholic, and cholic acid are constructed with one, two, and three QAC respectively, as cationic head groups via the hydroxyl functionality. This provides a unique avenue for tuning amphiphilicity and testing the level of facial amphiphilicity.

## Results

### Synthesis of cationic multicyclic natural product-derived polymers

**Fig. 3:**
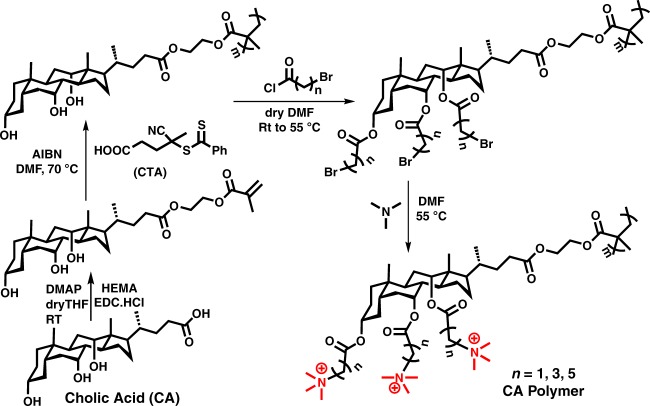
Synthesis of cholic acid-containing polymers. Cholic acid (CA) converted into (2-methacryloyloxy)ethyl cholate (MAECA); RAFT polymerization of MAECA; post-polymerization modification with bromoalkanoyl chloride; quaternization with trimethyl amine

**Fig. 4:**
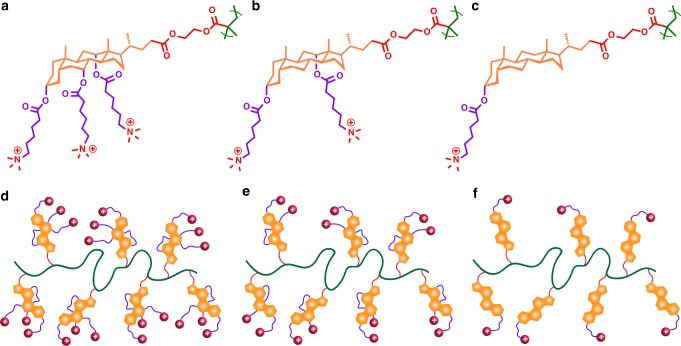
Multicyclic natural product-based cationic polymer structures and their illustration. **a**, **d** CA polymer, **b**, **e** DCA polymer, **c**, **f** LCA polymer

A class of cationic polymers was synthesized from bile acid derivatives in four steps. Methacrylate monomers of cholic acid, (2-methacryloyloxy)ethyl cholate (MAECA), deoxycholic acid, (2-methacryloyloxy)ethyl deoxycholate (MAEDA), and lithocholic acid, (2-methacryloyloxy)ethyl lithocholate (MAELA) were synthesized by simple esterification coupling reactions of respective bile acid and hydroxyethyl methacrylate (HEMA) at room temperature^45^. The reaction scheme in Fig. 3 illustrates the synthesis using cholic acid as an example. Each monomer of MAECA, MAEDA, and MAELA was then polymerized via reversible addition-fragmentation chain transfer (RAFT) polymerization utilizing 4-cyano-4-(thiobenzylthio)pentanoic acid as a chain transfer agent. Molecular weight of all three bile acid-containing polymers was controlled with low dispersity as determined by Gel Permeation Chromatography (GPC) (Supplementary Table 1). Hydroxyl groups of these homopolymers were further modified through an esterification reaction with bromoalkanoyl chloride. After post modification, the peaks next to the alcohol group in ^1^H NMR at ~ 3.2–3.8 ppm shifted to 4.7–5.2 ppm (Supplementary Figure 2a). The methylene group next to the bromine group appears at ~3.4–3.6 ppm, indicating the formation of an ester linkage. The disappearance of a broad peak at 3500–3600 cm^−1^ corresponding to hydroxyl groups and appearance of a higher intensity peak at 1720 cm^−1^ in FTIR spectra (Supplementary Figure 2b) for the ester group further confirmed the post-polymerization modification of hydroxyl groups in homopolymers. Evidence of successful post-polymerization modification was also established by the slight shift of GPC traces of polymers before and after modification (Supplementary Figure 2c). Finally, the bromine groups were substituted by trimethylamine to offer quaternary ammonium-containing polymers. The appearance of an intense peak at ~3.0 ppm for three methyl and one methylene group in ^1^H NMR spectra confirmed the formation of quaternary ammonium-containing polymers (Supplementary Figure 2a). Finally, cationic homopolymers with single, double, and triple QAC head groups were obtained from lithocholic acid, deoxycholic acid, and cholic acid respectively (Fig. 4).

Cholic acid-based cationic polymers having a series of molecular weight were further prepared to study the effect of molecular weight on antimicrobial activity (Supplementary Table 1). The spacer length of methylene (one, three, and five) between QAC and the ester group was also investigated in order to examine its effect on antimicrobial efficacy, for which polymers with similar molecular weight were used for post-polymerization modification. Polymers were denoted according to their respective derivative resource, molecular weight, and spacer unit (i.e. CA_19k_5 is a cholic acid polymer with molecular weight of 19,000 *g* mol^−1^ and a spacer of five methylene). To compare the antimicrobial activity with polymers, a three QAC-containing cholic acid-based monomer (labeled as CA_Monomer) was also prepared, shown in Supplementary Figure 1b. The experimental details are given in the Methods and Supplementary Information.

### Antimicrobial activities

The antimicrobial activities of multicyclic natural product-based cationic polymers were evaluated against clinically relevant Gram-positive bacteria *S. aureus* and Gram-negative bacteria *E. coli* and *P. aeruginosa*. Initially, the antimicrobial activity of three different bile acid polymers with a spacer of five methylene and molecular weight ~19,000 g mol^−1^ was evaluated by standard agar disc diffusion assay. The observation of clear inhibition zones indicated that all three polymers have potent activity against both Gram-negative and Gram-positive bacteria at different levels. Among them cholic acid polymers are the most effective, and lithocholic acid ones are the least (Supplementary Figure 8). Interestingly, the initial studies also revealed that all these polymers had higher efficacies towards Gram-negative bacteria than Gram-positive pathogens.

We then determined the minimum inhibitory concentration (MIC) of polymers by a broth microdilution method and compared the killing efficiency of each bile acid polymer. The MIC results (Table 1) demonstrated that the cholic acid-based polymer (CA_19k_5) exhibited more potent antimicrobial activity, with significantly lower MICs in comparison to deoxycholic acid (DCA_19k_5) and lithocholic acid (LCA_20k_5) based polymers. A delicate balance of hydrophobicity and hydrophilicity is one of the essential factors for selective interactions with bacterial membranes. Since all bile acid-based cationic polymers contain the same hydrophobic four fused rings in each repeating unit, the change in hydrophilicity is critical for the antimicrobial activities. The cholic acid-based polymer contains three QAC groups in each repeating unit, making it more hydrophilic with higher charge densities, whereas deoxycholic acid and lithocholic acid are less hydrophilic because of fewer charged groups. Consequently, deoxycholic acid-containing polymer DCA_19k_5 exhibited moderate sensitivity towards bacteria while LCA_20k_5 is least effective towards bacteria. Our results demonstrated that the higher charge densities of a polymer could lead to more significant interactions with bacterial membranes, similar to observations made by Yang and colleagues^46,47^. Though all polymers can inhibit bacterial growth, they again exhibited enhanced potency towards Gram-negative bacteria. For example, the MICs of CA_19k_5 are 11.2 and 3.1 μg mL^−1^ against *E. coli* and *P. aeruginosa* respectively, whereas about 19.1 μg mL^−1^ against *S. aureus*. This is significant as there are few antibiotics available for the treatment of infections by Gram-negative bacteria, in particular, pathogenic *P. aeruginosa*.

**Table 1:** Antimicrobial activity of different multicyclic natural product-based cationic polymers by a broth microdilution method

Polymers	Minimum inhibitory concentration (MIC)^a^ (µg mL^−1^)				HC_50_ (µg mL^−1^)	Selectivity of *E. coli* (ATCC-11775) (HC_50_/MIC)	Selectivity of *P. aeruginosa* (ATCC-10145) (HC_50_/MIC)	Selectivity of *E. coli* (ATCC-BAA-197) (HC_50_/MIC)
	*E. coli* (ATCC-11775)	*P. aeruginosa* (ATCC-10145)	*E. coli* (ATCC-BAA-197)	*S. aureus* (ATCC-33591)				
CA_19k_5	11.2	3.1	11.5	19.1	>306	>27	>98	>26
DCA_19k_5	11.5	6.4	20.4	24.6	>37	>3	>5	1
LCA_20k_5	11.4	3.4	20.5	56.8	NT	NT	NT	NT
*Effect of the spacer length on antimicrobial activity of cholic acid-based cationic polymers*								
CA_19k_5	11.2	3.1	11.5	19.1	>306	>27	>98	>26
CA_19k_3	12.5	10.4	12.4	19.6	>31	>2	3	2
CA_19k_1	25.6	22.2	37.7	45.6	>8	NT	NT	NT
*Effect of molecular weight on antimicrobial activity of cholic acid-based cationic polymers*								
CA_10k_5	6.4	3.0	6.8	15.3	>110	>17	>37	>16
CA_19k_5	11.2	3.1	11.5	19.1	>306	>27	>98	>26
CA_25k_5	11.4	10.5	11.9	19.1	>315	>28	>30	>26
CA_32k_5	12.2	19.4	14.5	27.4	>1886	>154	>97	130
CA_Monomer	22.3	12.8	22.5	25.6	NT	NT	NT	NT

*NT*  not tested

^a^MIC is the lowest polymer concentration that completely inhibits bacterial growth

**Fig. 5:**
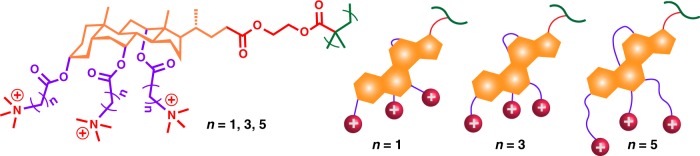
Cholic acid-based cationic polymers with different spacers. Chemical structures of polymers and their illustration

We further studied the effect of methylene spacers in cholic acid-based polymers on antimicrobial activity (Fig. 5). We observed that polymers containing a longer spacer showed more potent killing efficacy compared to those with shorter spacers. As shown in Table 1, CA_19k_5 polymer (5 methylene units separated from the cationic charge) exhibited higher antimicrobial activity against both Gram-positive and Gram-negative bacteria than CA_19k_3 and CA_19k_1. According to the snorkeling effect in peptides^48–50^, a longer spacer unit could provide increased hydrophobicity, and the additional distance between the QAC groups and the hydrophobic multicyclic ring attached to the polymer backbone would facilitate a deeper insertion of the polymer chain into the bacterial membrane. In contrast, a shorter spacer has less flexibility and room for extending the charge group through the membrane^51^. A longer spacer could not only facilitate the charge group easier to reach a target substrate (here cell membrane), but provide a flexible anchoring on surfaces without requiring a configurational change of the bulky triterpene structure.

Next, we explored the effect of molecular weight (*M*_n_) of polymers on the antimicrobial activity (Table 1). In case of *P. aeruginosa*, the MICs of higher molecular weight polymers increased. For *E. coli*, the molecular weight at the test range has a minimal effect on the activity. In case of Gram-positive bacteria, *S. aureus*, the lower molecular weight polymer CA_10k_5 exhibited a MIC of ~ 15.3 μg mL^−1^, whereas the MIC for CA_32k_5 was at 27.4 μg mL^−1^. These results indicated that CA polymer with ~10,000 g mol^−1^ molecular weight exhibited better efficacy than the higher molecular weight polymers. This could be explained by the potential trapping of higher molecular weight polymers in the dense, outmost peptidoglycan layer of *S. aureus*. This observation is consistent with the sieving effect, as also identified by Lienkamp et al^52^. We also evaluated the antimicrobial activity of a cationic cholic acid based monomer (CA_Monomer, Table 1), which is lower than that of polymers. This might be due to the increase of the density of local facial amphiphilicity from polycations than monomers, which was similarly observed by many other groups on different systems^53^.

Antimicrobial activity was further investigated using a clinically isolated MDR strain of *E. coli* (ATCC-BAA-197). As shown in Table 1, all cholic acid polymers containing a five-methylene spacer inhibited the growth of this strain, and with low MIC values (7–15 μg mL^−1^), demonstrating a high efficacy against MDR *E. coli*. These MIC values increased with polymers containing the shorter spacer unit. However, the MIC values are comparatively higher than those for regular strains of *E. coli* (ATCC-11775), which is possibly due to varying phospholipid compositions. It is worth noting that the cholic acid polymers with a molecular weight in the range of 10,000–20,000 g mol^−1^ is also more efficient at inhibiting bacterial growth than those with higher molecular weight.

**Fig. 6:**
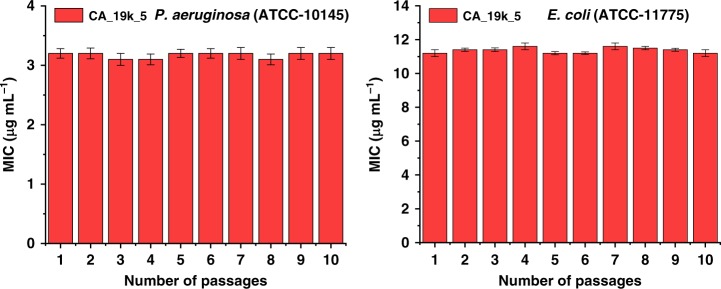
Drug resistance study of CA_19k_5 against *P. aeruginosa* and *E. coli* upon multiple sublethal dose treatment. The Data are collected from the three replicates and the error bars represent the s.d. of three replicates

To evaluate the possible bacterial resistance of cholic acid-based polymers, we performed an antimicrobial resistance study for one of the most potent polymers, CA_19k_5, against *P. aeruginosa* and *E. coli*. Bacteria were exposed multiple times to the polymer at a sub-MIC level, and the MIC was measured for every consecutive passage. Detailed experimental procedures are provided in the supplementary information. After 10 passages, no significant changes in the MIC values were observed, as detailed in Fig. 6. This important result demonstrated that developing resistance against cholic acid-based cationic polymers is inherently difficult for both *P. aeruginosa* and *E.coli* bacterial strains.

### Hemolytic activities

The toxicity of bile acid-derived cationic polymers was evaluated by measuring hemoglobin release from mouse red blood cells (RBCs) at various concentrations. The selectivity for bacterial cells over mammalian cells was determined by the ratio of HC_50_ (the concentration of a polymer that causes 50% hemolysis of RBCs) to MIC values (HC_50_/MIC). As mentioned previously, the hydrophobic and hydrophilic balance of an antimicrobial polymer plays a critical role for the selective attachment to a bacterial cell membrane. It is well established that a polymer with higher hydrophobicity or lower hydrophilicity produces hemolysis to a greater extent, due to the strong interaction with the lipid portion of a mammalian cell membrane^25,47,54^. As shown in Table 1, all cholic acid polymers exhibited negligible hemolysis at their respective MIC values, demonstrating excellent selectivity toward a broad range of pathogenic microbes over mammalian cells.

Bile acid derivatives are intrinsically hydrophobic due to the presence of a four fused-ring structure. All cholic acid polymers contain three positive head groups in each repeat unit, which reduces hydrophobicity. In contrast, the deoxycholic acid-based polymer possesses only two positive charged head groups in each repeat unit, making it more hydrophobic with a substantial level of toxicity. The hemolysis activity of lithocholic acid-based cationic polymers was not determined due to poor solubility in water. Additionally, the molecular weight of polymers was also found to have some effect on hemolysis activity (Table 1). We observed that HC_50_ increased with the increase of molecular weight of cholic acid-based polymers. The length of spacers also has an enormous impact on hemolysis, as shown in Table 1. We observed that the cholic acid polymers containing shorter spacers (CA_19k_1 and CA_19k_3) are more toxic compared to the longer spacer containing polymer (CA_19k_5). There are many parameters to influence the hemolytic activity, especially the balance of hydrophilicity and hydrophobicity. The low HC_50_ value for CA_19k_1 might be related with insufficient electrostatic interactions due to the short spacer linking cationic charges, which could amplify the hydrophobic effect by cholic acid on the more hydrophobic nature of membranes from mammalian cells.

### Mechanisms of action

**Fig. 7:**
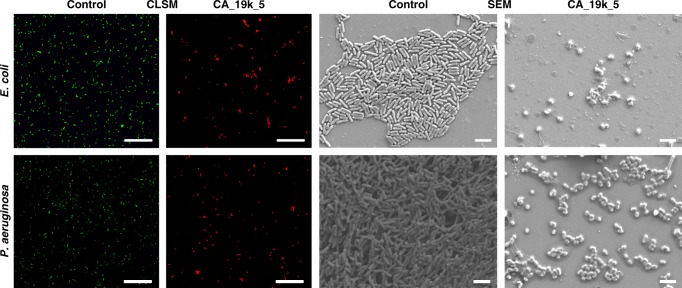
CLSM and SEM images of *E. coli* and *P. aeruginosa* under control and CA_19k_5 treatment with two times of MIC concentration. Bacteria concentrations were 1.0 × 10^6^ CFU/mL. Bacterial solutions without CA_19k_5 were used as the control. Scale bar in confocal images is 25 µm and scale bar in SEM images is 2 µm

To elucidate the mode of action of bile acid-derived polymers against bacteria, we performed confocal laser scanning microscopy (CLSM) to investigate the membrane permeability changes before and after treatment with CA_19k_5 polymer using a LIVE/DEAD BacLight assay kit. The concentration of polymers is two times that of the MIC value. As shown in Fig. 7, green colored cells were observed for control bacteria (*E. coli* and *P. aeruginosa*), revealing most cells live with intact bacterial membranes. In contrast, when the bacteria were treated with polymer CA_19k_5, most cells were killed. These findings revealed that the antimicrobial activity of bile acid-based cationic polymers occurred by the disruption of bacterial membrane, consistent with the membrane lytic mechanism of various synthetic antimicrobial polymers^28,39,46,55^. In case of *S. aureus*, these polymers are less effective (Supplementary Figure 9). The antimicrobial mechanism of action was further investigated through the observation of morphological changes of bacterial cells after CA_19k_5 polymer treatment using scanning electron microscopy (SEM). Bacteria *E. coli* and *P. aeruginosa* under control remained intact with smooth surfaces as shown in Fig. 7, whereas polymer-treated cells were significantly damaged and highly distorted from the original morphology. Most bacterial cells were shown to be significantly fragmented. The significant physical damage of cell membranes was observed for *S. aureus* only when the concentration of polymers was increased to four times that of the MIC value (Supplementary Figure 10). The loss of original morphology with cell membrane damage was more apparent in the case of Gram-negative bacteria compared to that of Gram-positive bacteria.

## Discussion

Bile acid derivatives (mostly small molecules) have been developed as antimicrobial agents. Moore et al.^56^ reported that cationic bile salts share some structural features with an antibiotic squalamine isolated from sharks. Diamond et al. prepared a family of head-to-tail cationic lipids that combine cholic acid and spermine, which showed enhanced antimicrobial activity related to increased hydrophobicity, although no facial amphiphilicity was explored^57^. Savage and co-workers claimed that membrane-active facial amphiphilic cationic molecules, such as bile acid derivatives, could disrupt bacterial membranes^58,59^. Cholic acid-derived cationic surfactants can form micellar structures that exhibit antimicrobial activity against Gram-positive and Gram-negative bacteria^60^. However, higher susceptibility to the resistance of these small molecules remains a significant issue.

In the current study, we developed a class of antimicrobial polymers from bile acids, which possess macromolecular conformations critical for interactions with bacterial membranes. We observed that cholic acid-based cationic polymers are more effective against Gram-negative bacteria, especially *P. aeruginosa*, than Gram-positive bacteria (e.g. *S. aureus*). Different from Gram-positive bacteria using peptidoglycan as the major periphery enveloping their cell membranes, Gram-negative bacteria possess double membranes with the outer membrane made up of zwitterionic phosphatidylethanolamine (PE) and other anionic phospholipids as their periphery for self-defense. Therefore, in Gram-negative bacteria it is more challenging for antimicrobial agents to balance their hydrophobicity and cationic charges as well as to adopt a conformation that is favorable for interactions with the outer membrane.

**Fig. 8:**
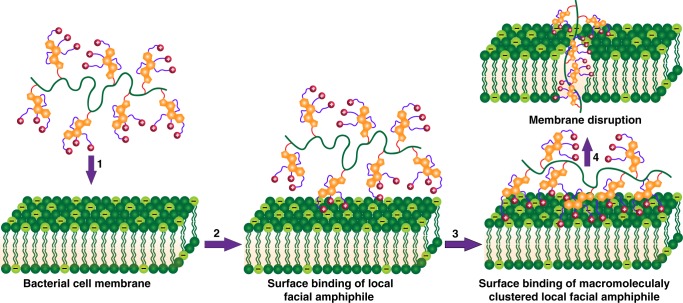
A proposed mechanism of action of cholic acid-based polymers on the bacterial cell membrane: 1 diffusion, 2 surface binding, 3 membrane insertion and 4 membrane disruption. The illustrated cholic acid can be replaced by other multicyclic compounds that are modified with facial amphiphilicity

The hydrophobic multicyclic structure and three oriented cationic charges in the modified cholic acid provide true facial amphiphilicity in contact with bacterial cell membranes. Initially, three cationic charges on each cholic acid unit localize onto the outer membrane as a result of electrostatic interactions (Fig. 8), then the hydrophobic face of cholic acid inserts into the membrane. Since each of this unique moiety is attached to a flexible macromolecular chain, collectively tens of (or even hundreds of) these local facial amphiphilic structures would facilitate each other and promote the entire macromolecule to penetrate through the membrane (Fig. 8). Such a concerted penetration of macromolecular chains across the cell membrane would cause its destabilization and fragmentation, ultimately leading to cell death. With this design, there is no need for an entire macromolecule to adopt a globally entropy-unfavorable facial amphiphilic conformation. Conversely, Gram-positive bacteria, like *S. aureus*, have membranes primarily composed of anionic lipids such as phosphatidylglycerol (PG) and cardiolipin (CL), which is overlain by a dense and thick peptidoglycan layer^46^. Bulky cholic acid-based polymers could be more easily trapped in this layer and thus less effective in disrupting these cell membranes.

In summary, we reported a design of antimicrobial polymers with repeat units possessing local facial amphiphilicity, which could promote effective interactions of an entire macromolecule with bacterial cell membranes, circumventing the adoption of an energetically unfavorable global facial amphiphilicity. Specifically, we derivatized three different multicyclic natural products. Among them, cholic acid polymers were shown to be more efficient than their deoxycholic and lithocholic acid counterparts, regarding both antimicrobial activity and selectivity. This is ascribed to the true facial amphiphilic structure from cholic acid derivatives, which have the hydrophobic multicyclic structure as one face and three oriented hydrophilic cationic charges as the other face. It is worth noting that a lot of multicyclic natural and synthetic compounds could be used as the key building block. This macromolecular structure and conformation may open an avenue toward next-generation antimicrobial agents to treat multidrug-resistant Gram-negative bacteria.

## Methods

### Synthesis of monomers

(2-methacryloyloxy)ethyl cholate (MAECA) monomer was synthesized via an esterification reaction between CA and 2-hydroxyethyl methacrylate (HEMA) in the presence of 1-(3-dimethylaminopropyl)-3-ethyl carbodiimide hydrochloride (EDC·HCL) and 4-dimethylamino pyridine (DMAP). Initially, CA (5.0 g, 12.24 mmol) and DMAP (0.16 g, 1.35 mmol) were dissolved in 40 mL of dry tetrahydrofuran (THF) under nitrogen. EDC·HCl (2.58 g, 13.46 mmol) was added to the solution. After placing the reaction mixture in an ice bath, HEMA (1.75 g, 13.56 mmol) was added dropwise to the solution and then progressed for 48 h at room temperature. The reaction mixture was filtered and evaporated. The crude product was redissolved in dichloromethane (DCM) (60 mL) and washed with 5% HCl solution (25 mL × 1), saturated NaHCO_3_ (25 mL × 3), water (25 mL × 2), and brine solution (25 mL × 2). After drying the organic layer over anhydrous MgSO_4_, the solvent was removed by rotary evaporation. Silica column chromatography with hexane: ethyl acetate (7: 3) as eluents was carried out to yield a product with a yield of 60%. ^1^H NMR (Supplementary Figure 2a) (300 MHz, CDCl_3_, *δ*, ppm): 6.13 and 5.59 (2 H, s, a), 4.33 (4 H, m, b & b’), 3.96 (1 H, t, c), 3.84 (1 H, q, d), 3.45 (1 H, m, e), 1.94 (3 H, s, f), 0.97 (3 H, d, g), 0.88 (3 H, s, h), and 0.67 (3 H, s, i). ES-MS (Supplementary Figure 11): observed *m*/*z* for [M + Na^+^] 543 and [M + H^+^] 521.

(2-methacryloyloxy)ethyl deoxycholate (MAEDA) was synthesized according to a similar procedure to the synthesis of MAECA. ^1^H NMR (Supplementary Figure 3a) (300 MHz, CDCl_3_, *δ*, ppm): 6.13 and 5.59 (2 H, s, a), 4.33 (4 H, m, b & b’), 3.97 (1 H, t, c), 3.61 (1 H, m, d), 1.94 (3 H, s, e), 0.97 (3 H, d, f), 0.88 (3 H, s, h), and 0.67 (3 H, s, g). ES-MS (Supplementary Figure 12): observed *m*/*z* for [M + Na^+^] 527 and [M + H^+^] 505.

(2-methacryloyloxy)ethyl lithocholate (MAELA) was also synthesized according to a similar procedure to the synthesis of MAECA, except for the purification process. Silica column chromatography with hexane: ethyl acetate (3: 2) as eluents was carried out to yield a product with a yield of 50%. ^1^H NMR (Supplementary Figure 3b) (300 MHz, CDCl_3_, *δ*, ppm): 6.12 and 5.59 (2 H, s, a), 4.33 (4 H, m, b & b′), 3.61 (1 H, m, c), 1.94 (3 H, s, d), and 0.62 (3 H, s, e). Direct-probe mass spectrum (Supplementary Figure 13): observed *m*/*z* 488.

### Synthesis of bile acid polymers

Methacrylate monomers were polymerized using a typical RAFT polymerization technique^45^. For example, MAECA (0.70 g, 1.35 mmol), 4-Cyano-4-(thiobenzylthio)pentanoic acid (CTP) (6.27 mg, 0.0224 mmol), and azobisisobutyronitrile (AIBN) (0.74 mg, 4.487 µmol) were placed in a 10 mL Schlenk flask and dissolved in *N*, *N*-dimethylformamide (DMF) (2 mL). The mixture was performed with three freeze-pump-thaw cycles protected under nitrogen and immersed into a preheated oil bath set at 70 °C. After a certain period of time, the polymerization was quenched by exposure to air and cooled under an ice water bath. The reaction mixture was precipitated twice into a mixture of hexane and DCM (50:50) and finally dissolved in THF and precipitated into hexane. The polymer was dried under vacuum.

### Synthesis of bromoalkyl-containing bile acid polymers

CA polymer (300 mg) was placed in a 25 mL round bottom flask and dissolved in anhydrous DMF (3 mL). Excess 6-bromohexanoyl chloride (3 mL) or 4-bromobutanoyl chloride (3 mL) or bromoacetyl bromide (3 mL) was added to the polymer solution dropwise at room temperature. The reaction mixture was allowed to stir at 55 °C for 48 h and precipitated into methanol. The product was redissolved in DCM (2 mL), precipitated in methanol twice, and dried under vacuum. The reaction was confirmed by ^1^H NMR and FTIR. Similarly, DCA and LCA polymers were modified. ^1^H NMR spectra of post-modified CA, DCA, and LCA polymer with 6-bromohexanoyl chloride are shown in Supplementary Figures 2a, 3a, and b respectively. FTIR spectra of modified CA, DCA, and LCA polymers with 6-bromohexanoyl chloride are shown in Supplementary Figures 2b, 4a, b respectively. ^1^H NMR spectra of modified CA polymer with 4-bromobutanoyl chloride and bromoacetyl bromide are shown in the Supplementary Figure 5a, b respectively.

### Synthesis of QAC-containing polymers

As an example: 6-bromohexyl-modified CA polymer (300 mg) was dissolved in DMF (4 mL). Then, trimethylamine solution (33 wt%, 9 mL) in ethanol was added to the reaction mixture and stirred for 24 h at 55 °C. After cooling and concentrating the reaction mixture, the resulting solution was precipitated in THF and centrifuged to collect the product. The product was washed with THF and dried under vacuum. Finally, the product was further purified by dialysis against DI water (1 L × 3) for 24 h. The solution in dialysis bag was collected and freeze-dried to obtain a white product. DCA and LCA polymers were similarly quaternized.

### Synthesis of QAC-containing CA monomer

MAECA (0.50 g, 0.96 mmol), triethyl amine (2.91 g, 28.84 mmol), hydroquinone (0.19 mmol, 0.021 g), and catalytic DMAP (0.035 g, 0.29 mmol) were dissolved in dry THF (10 mL) under nitrogen. Then, 6-bromohexanoyl chloride (2.59 g, 14.42 mmol) was added dropwise to the mixture at 0 °C was then stirred at room temperature for 36 h. The reaction mixture was filtered and evaporated. The residue was diluted with DCM and washed with water (3 times), saturated NaHCO_3_ (3 times) and brine solution (one time). The organic phase was dried over magnesium sulfate and concentrated, then precipitated in hexane twice to remove unreacted 6-bromohexanoyl chloride. The product was further purified by the silica column chromatography with hexane: ethyl acetate (1: 4) as eluents to obtain a product with a yield of 55%. The yellow product was dried under vacuum. The reaction was confirmed by ^1^H NMR (Supplementary Figure 6) and FTIR (Supplementary Figure 7). ^1^H NMR (300 MHz, CDCl_3_, *δ*, ppm): 6.13 and 5.59 (2 H, s, e), 5.18 (1 H, t, a), 5.01 (1 H, q, b), 4.53 (1 H, m, c), 4.29 (4 H, m, d & d′), 3.5 (6 H, t, g, g′& g′′), 2.48 (6 H, t, h, h′ & h′′), and 1.91 (3 H, s, f).

Compound **1** (200 mg, 0.19 mmol) (Supplementary Figure 1b) was dissolved in DMF (3 mL). Then, trimethylamine solution (33 wt%, 10 mL) in ethanol was added to the reaction mixture and stirred for 24 h in a closed reaction vessel at 55 °C. After cooling and concentrating the reaction mixture, the resulting solution was precipitated in THF and centrifuged to collect the product. The product was further washed with THF and dried under vacuum. The reaction product was confirmed by ^1^H NMR (Supplementary Figure 6).

## Electronic supplementary material

Supplementary Information

Reporting Summary

## Data Availability

All the data supporting the findings of this study are available within the Article and its Supplementary Information file.
