# Supplementary material for: Macromolecular-clustered facial amphiphilic antimicrobials
Source: Nat Commun. 2018 Dec 7;9:5231. doi: 10.1038/s41467-018-07651-7 (PMC6286373; doi:10.1038/s41467-018-07651-7)
Supplement: Supplementary file 1 — Supplementary Information [file 41467_2018_7651_MOESM1_ESM.pdf]

Supplementary Information

Macromolecular-Clustered Facial Amphiphilic Antimicrobials

Rahman et al.

Supplementary Methods

Materials. All chemicals were purchased from commercial sources and used as received unless otherwise stated. Cholic acid (CA, ≥98%), deoxycholic acid (DCA, ≥98%), lithocholic acid (LCA, ≥98%), 2-hydroxyethyl methacrylate (HEMA, 97%), and 4-dimethylamino pyridine (DMAP, 99%) were purchased from Sigma-Aldrich. 1-(3-dimethylaminopropyl)-3-ethyl carbodiimide hydrochloride (EDC·HCl, 98%) was purchased from TCI. 6-Bromohexanoyl chloride (97%), 4-bromobutanoyl chloride (97%), bromoacetyl bromide (98%) and trimethylamine (33% w/w in ethanol denatured with 2% cyclohexane) was purchased from Alfa-Aesar. 4-Cyano-4-(thiobenzylthio)pentanoic acid (CTP, 97%) was purchased from Strem Chemicals Inc. Azobisisobutyronitrile (AIBN, Sigma, 98%) and solvents such as hexanes, anhydrous *N*, *N*-dimethylformamide (DMF, 99.9%), tetrahydrofuran (THF), dichloromethane (DCM), *etc.* were purified by standard procedures. $CDCl_3$ (99.9% D), D_2O (99.9% D) and DMSO-d_6 (99.9% D) were purchased from Cambridge Isotope Laboratories, Inc.

Characterization. The monomer and compound purity and polymer conversion were monitored by proton nuclear magnetic resonance 300 MHz (^{1}H NMR) spectroscopy using Bruker Avance III HD 300 spectrometer. Spectra were recorded in deuterated chloroform, Deuterium oxide or dimethylsulfoxide solvent in ppm (δ) with tetramethylsilane as an internal standard. Molecular weight and molecular weight distribution of polymers were measured by gel permeation chromatography (GPC) in THF equipped with a Waters 1525 Binary Pump, three Styragel columns, and a Waters 2414 Refractive Index (RI) detector. HPLC grade THF solvent was used as eluent at 35 °C with a flow rate of 1.0 mL min^{-1}. A series of narrowly-dispersed polystyrene standards obtained from Polymer Laboratories were used to calibrate the GPC system. GPC samples were prepared by dissolving polymers in HPLC grade THF at a concentration of 5-10 mg mL^{-1} and filtered by PTFE micro-filters with an average pore size of 0.2 μm.

Measurements of Antimicrobial Activity. The introduction of cationic charges to each polymer showed increased solubility in water. However, only cholic acid-based polymers are well soluble in water (~200 mg mL^{-1}). Deoxycholic acid polymers show limited solubility, while lithocholic acid polymers are not well soluble in water, due to the low charge density and high hydrophobicity. All polymers are well soluble in dimthyl sulfoxide (DMSO).

Bacteria Cell Culture. *Escherichia coli* (*E. coli*, ATCC-11775), *Pseudomonas aeruginosa* (*P. aeruginosa*, ATCC-10145), *Escherichia coli* (*E. coli*, ATCC- BAA-197), and *Staphylococcus aureus* (*S. aureus*, ATCC-33591) were purchased from ATCC. For these bacteria, a single colony was inoculated in 30 mL Tryptic Soy broth (TSB) at 37 °C for 24 h, shaking at 190 rpm min^{-1}. All bacteria were grown to an optical density of about l.00 (OD_{600} = 1.00) for further use.

Disk-diffusion Assays. The agar disk-diffusion assays were conducted by following literature.[1] At first, actively growing cultures of each bacterial strain on Mannitol salt agar (MSA) were inoculated on TSB agar plates. The bacterial growth culture (cell concentrations were 1.0×10^6 CFU/mL; 10 μL) was diluted to 1 mL in TSB solution. Subsequently, 100 μL of that bacterial solution was spread on TSB agar plates to form a bacterial lawn covering the plate surface. Then, 6 mm (diameter) filter discs were added to the plate surface. Each bile acid-containing polymer at different concentrations in DMSO was added to disks, and the plates were incubated at 28 °C for 18 h. The development of a clear zone around the disk is known as the inhibition zone, where bacteria are unable to grow. This inhibition zone indicates the ability of agents to kill bacteria.

Minimum Inhibitory Concentration (MIC) Measurements. The MIC of cationic bile acid-containing homopolymers and bile acid compounds were determined using a broth microdilution method.[2, 3] DMSO solution of homopolymer or compound with different concentrations was placed into each well of a 96-well plate. Different amount of TSB solution was added to antibiotic containing each well to get the volume of 150 μL. Then, 50 μL of bacterial TSB solution (OD_{600} = 0.07) was added into each well containing polymer solutions. The bacterial TSB solution without polymers or compounds was used as the control. The 96-well plate was incubated at 37 °C under constant shaking of 100 rpm for ~18 h until satisfactory growth. Bacterial growth was detected at OD_{600}, and was compared to controls of bacterial TSB solution without polymers. All assays were carried out in triplicates in the same assay plate. Optical density was plotted against polymer concentration, and linear regression analysis was used to determine the lowest concentration at which the optical density reading becomes zero. The MIC was taken as the concentration of bile acid-based polymers and compounds at which no microbial growth was observed.

Drug Resistance Study. A drug resistance study was performed against *P. aeruginosa* and *E. coli* for one of the active antimicrobial polymers named as CA_19k_5. Initially, the MIC of the polymer was measured as described above. The 10 μL bacterial solution was taken from the well that

contains polymer solution of 2×MIC and incubated for over night at 37 ^{o}C. Then, using this newly grown bacterial solution previously exposed to the polymer sample, the new MIC was determined. This assay was repeated for ten passes and MIC values were determined each time. Development of drug resistance was analyzed by observing the change in MIC after every pass. A polymer showing the same MIC in each successive passage indicates the bacteria did not develop resistance to the polymer.

Hemolysis Evaluation. Blood was collected from mice in heparinized tubes and diluted by mixing 800 μL of blood with 1000 μL of PBS. Polymer samples were prepared in PBS at concentrations of 0.5, 1, 2 4, 16, 31, 62, 125, 250, and 500 μg mL^{-1}, and 60 μL of the diluted blood samples was added to 3 mL of polymers, PBS, or 0.1% Triton-X100 in PBS. The samples were incubated for 1 h at 37 °C followed by centrifugation for 10 min at 1500 rpm. Supernatants were collected, and OD was measured at 545 nm to calculate hemolysis rate by using the equation, HR = (AS – AN)/(AP – AN), where AS, AN, and AP are OD values of the supernatants from test samples, negative control (PBS), and positive control (0.1% Triton-X100), respectively.

LIVE/DEAD Bacterial Viability Assays. Confocal laser scanning microscopy (CLSM) analysis was used to study the bacterial membrane permeability after polymer treatment. The bacterial strains were inoculated at 37 °C together with polymer CA_19k_5 at two times the MIC value following the same procedure for MIC determination. An untreated bacterial solution was used as controls. After 18 h incubation at 37 °C, 1 μL of LIVE/DEAD BacLight (Bacterial Viability Kit; Invitrogen Inc.) was added to 5 μL incubated solution and incubated for 15 min. The LIVE/DEAD BacLight bacterial viability kit consists of propidium iodide (PI), and FITC-labeled SYTO 9 dye used to stain nucleic acid (DNA). Green-fluorescing SYTO 9 can enter all cells, live or dead, whereas red fluorescing PI can only stain the DNA of damaged cytoplasmic membranes of dead or dying cells. Cells were imaged under a Leica TCS SP5 CLSM with a 63× oil immersion lens. When excited at 488 nm with argon and helium/neon lasers, bacteria with intact membranes display green fluorescence (emission = 500 nm), and bacteria with disrupted membranes fluoresce red (emission = 635 nm).

Bacterial Morphology Assays. The scanning electron microscopy (SEM) was used to examine morphologies of different bacteria with a similar procedure. In general, 10 μL of bacterial cell solution was grown on one glass slide in a 12-well plate containing 1 mL of TSB medium at 37

°C overnight. Cell suspensions were diluted to OD_{600} = 1.0. The polymer at twice the MIC was added to the 1 mL bacteria stock solution and incubated at 37 °C overnight. A bacterial solution without any polymers was used as the control. The samples were then fixed in cacodylate buffer with 2.5% glutaraldehyde solution (pH = 7.2) for 2–3 h at 4 °C and post-fixed with 1% osmium tetraoxide at 4 °C for 1 h. The samples were dried at a critical point, then coated with gold using a Denton Dest II Sputter Coater for 120 s, and observed by FE-SEM. An untreated cell suspension was used as the control.

Supplementary Figures

a

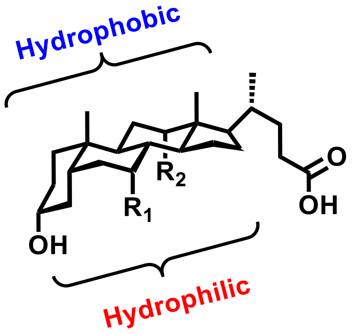

Bile Acid	R_1	R_2
Cholic Acid	OH	OH
Deoxycholic Acid	H	OH
Chenodeoxycholic Acid	OH	H
Lithocholic Acid	H	H

b

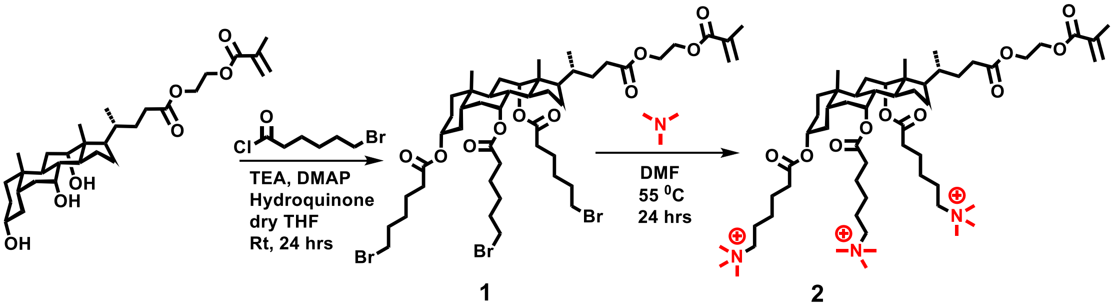

Supplementary Figure 1. Bile acid derivatives and cationic monomer synthesis. **a** Structures of bile acid derivatives; **b** Reaction scheme for the synthesis of cholic acid-containing QAC monomer (labeled as CA_Monomer).

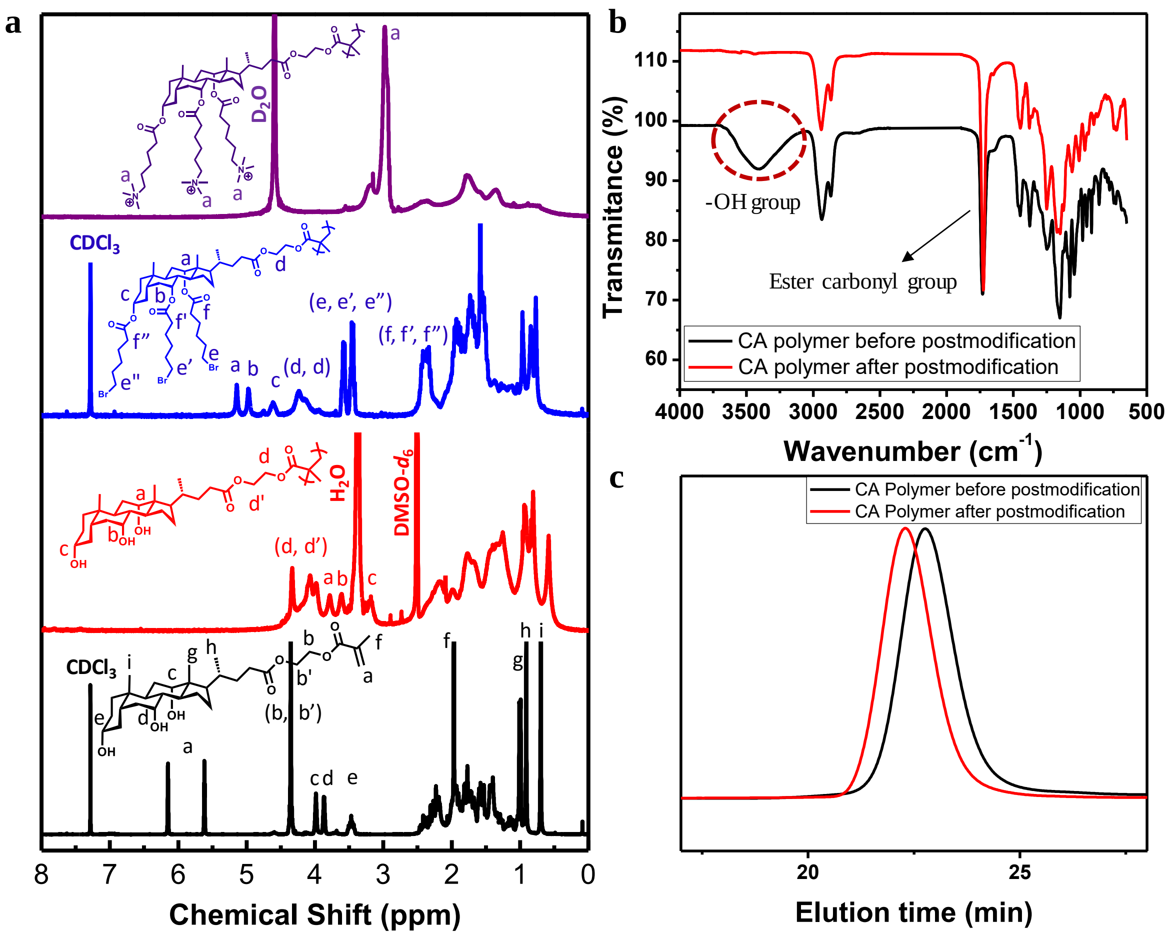

Supplementary Figure 2. Characterization of cholic acid polymer. **a** ^{1}H NMR spectra for cholic acid polymers; **b** FTIR spectra of cholic acid polymers (black) and polymers after post-polymerization modification (red); **c** GPC traces of the CA_19k_5 polymers before and after post-polymerization modification.

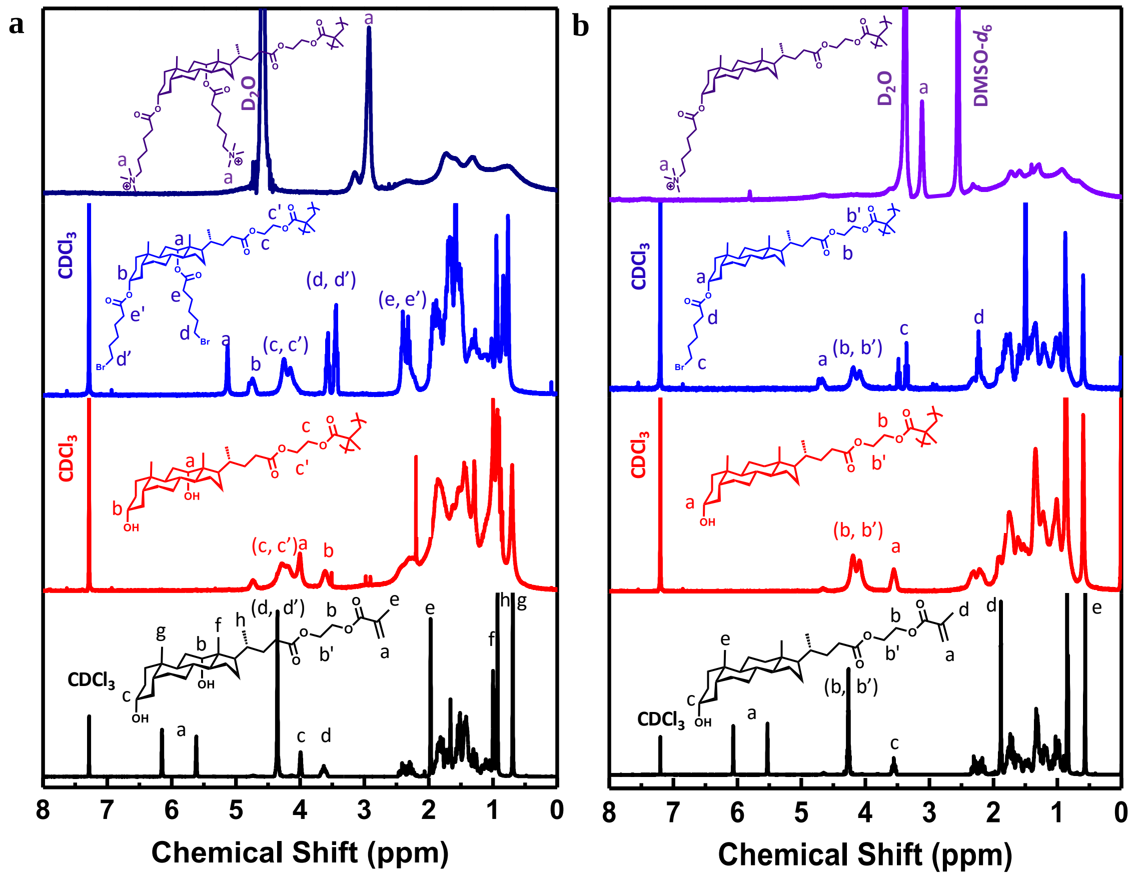

Supplementary Figure 3. ^{1}H NMR spectra of **a** Deoxycholic acid polymers; **b** Lithocholic acid polymers.

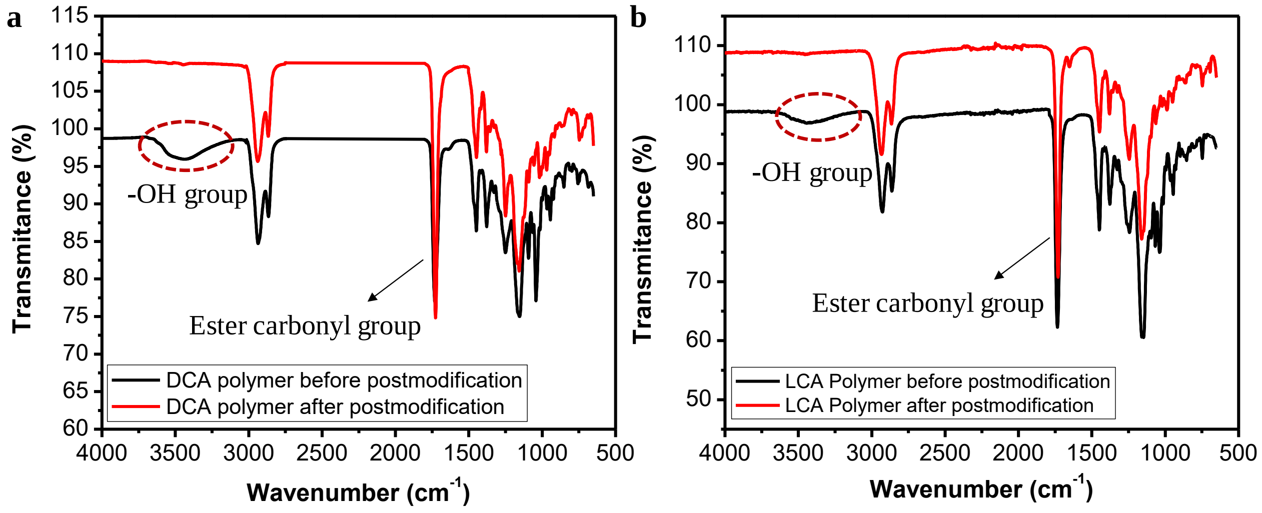

Supplementary Figure 4. FTIR spectra of **a** Deoxycholic polymers (black) and polymers after post-polymerization modification (red); **b** Lithocholic polymers (black) and polymers after post-polymerization modification (red).

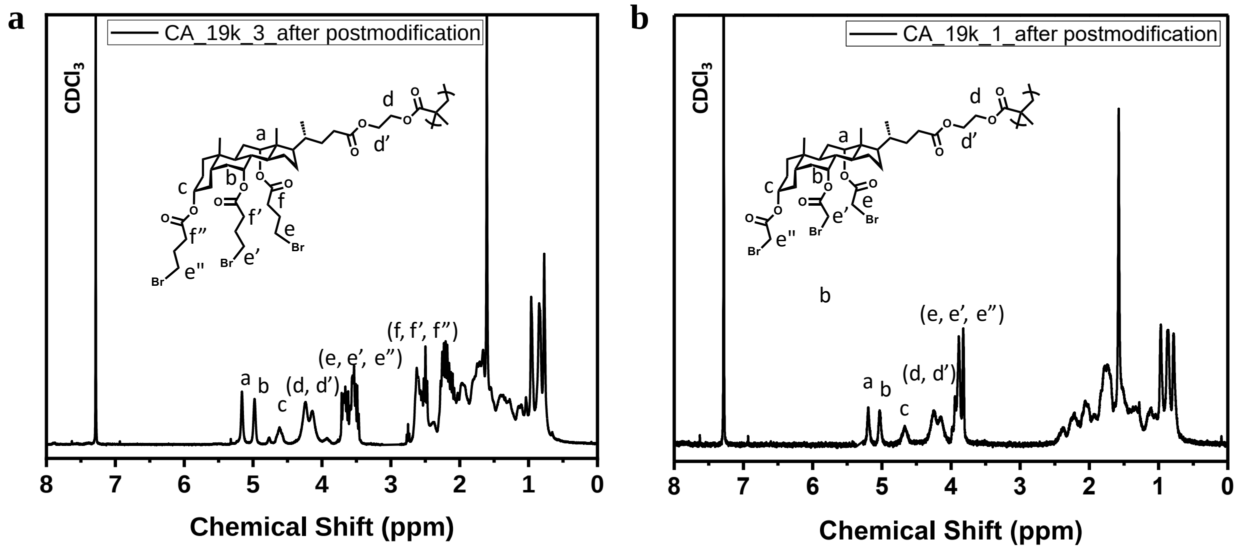

Supplementary Figure 5. ^{1}H NMR spectra for **a** the CA_19k_3 polymer after post-polymerization modification; **b** the CA_19k_1 polymer after post-polymerization modification.

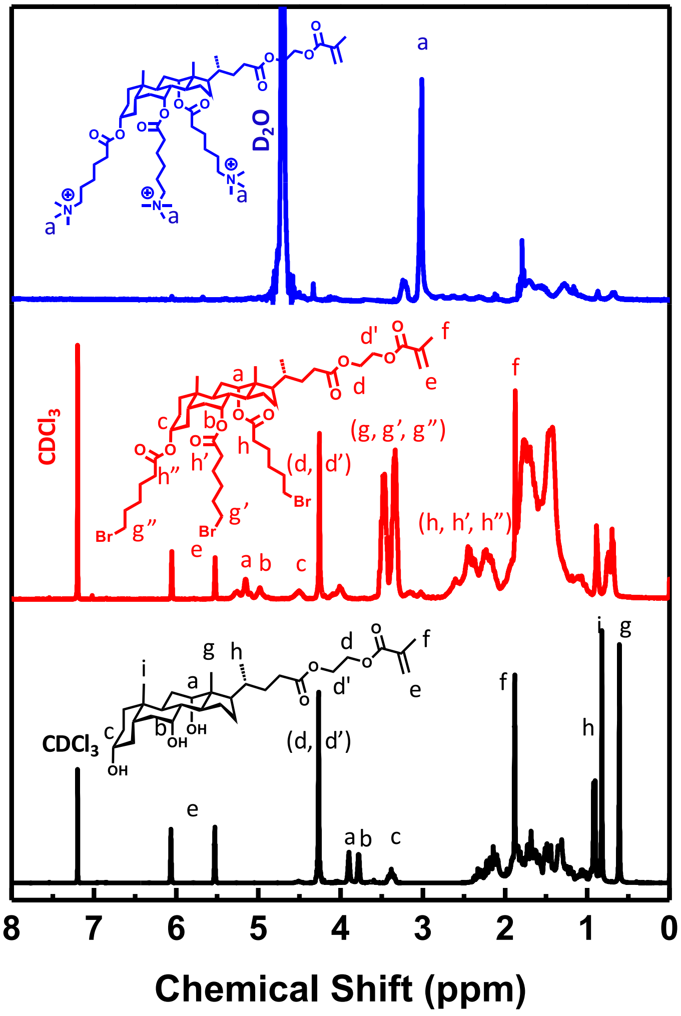

Supplementary Figure 6. ^{1}H NMR spectra of QAC-containing cholic acid monomer and its precursors.

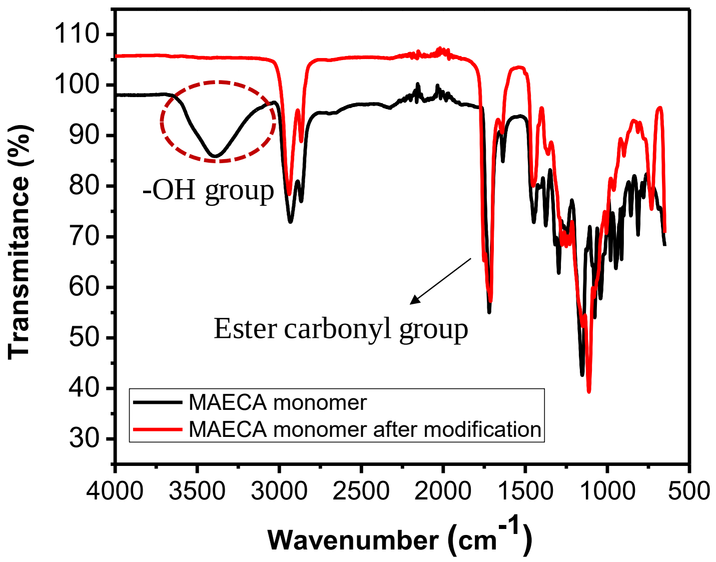

Supplementary Figure 7. FTIR spectra of MAECA monomer (black) and MAECA monomer after modification (red).

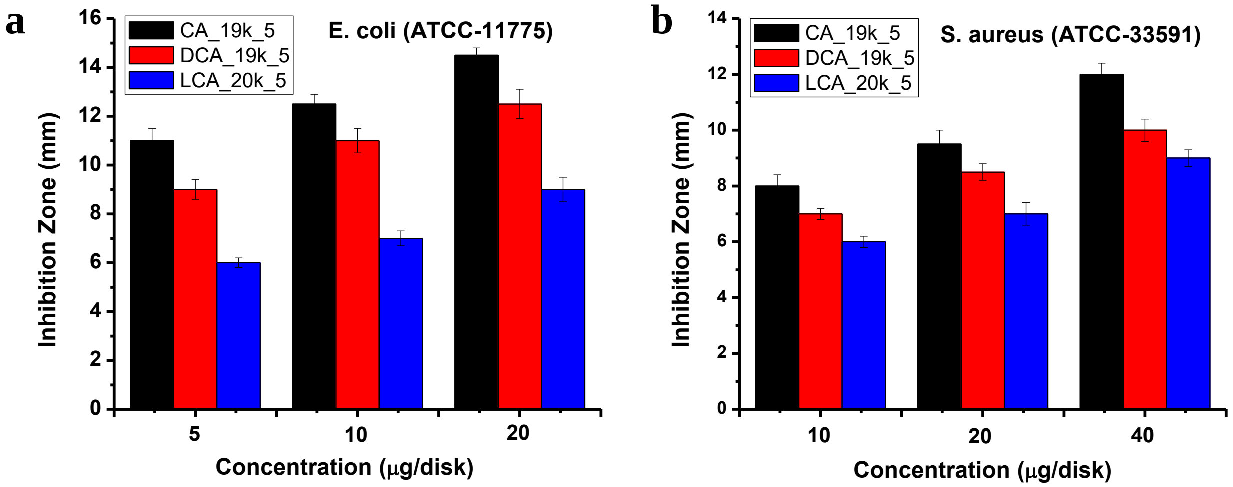

Supplementary Figure 8. Antimicrobial activities of polymers CA_19k_5, DCA_19k_5 and LCA_19k_5 as demonstrated by disk diffusion assay against **a** *E. coli* and **b** *S. aureus*. The error bars represent the s.d. of three replicates.

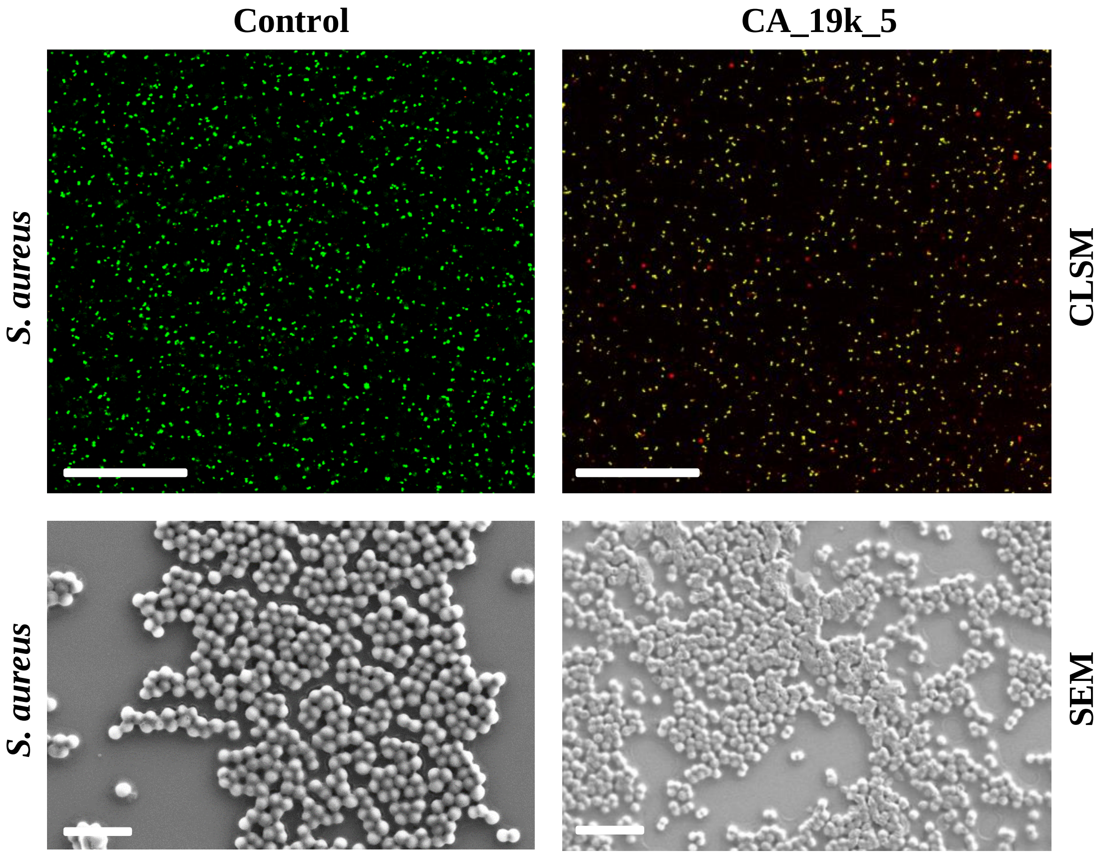

Supplementary Figure 9. CLSM and SEM images of control and CA_19k_5 polymer. CA_19k_5 treatment with two times of MIC concentration. Bacterial solutions without CA_19k_5 were used as the control. Scale bar in confocal images is 50 µm and scale bar in SEM images is 2 µm.

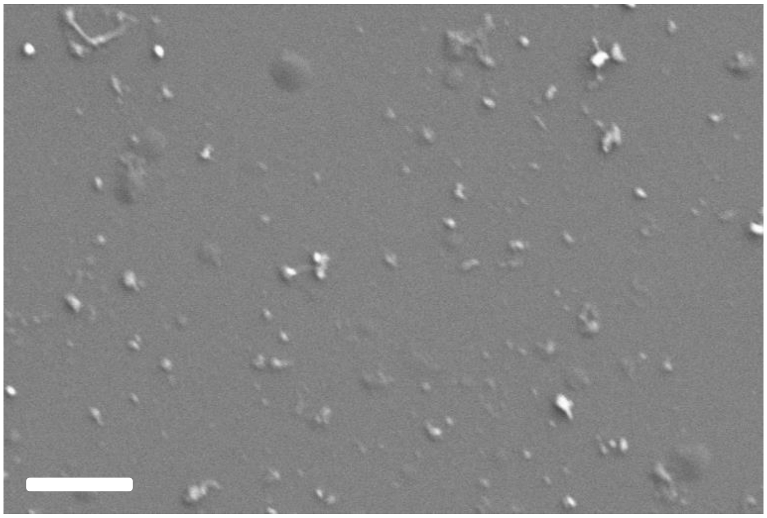

Supplementary Figure 10. SEM image of *S. aureus* with CA_19k_5 polymer treatment of four times MIC concentration. Scale bar in SEM images is 2 µm.

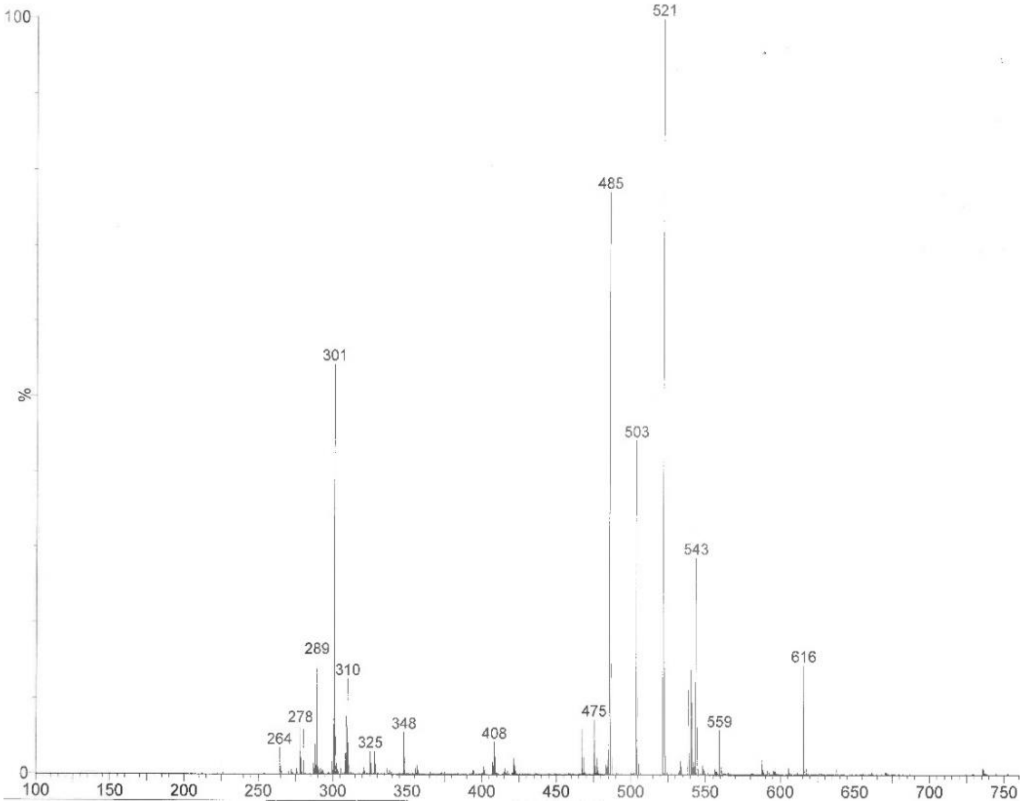

Supplementary Figure 11. Mass spectrum of MAECA monomer.

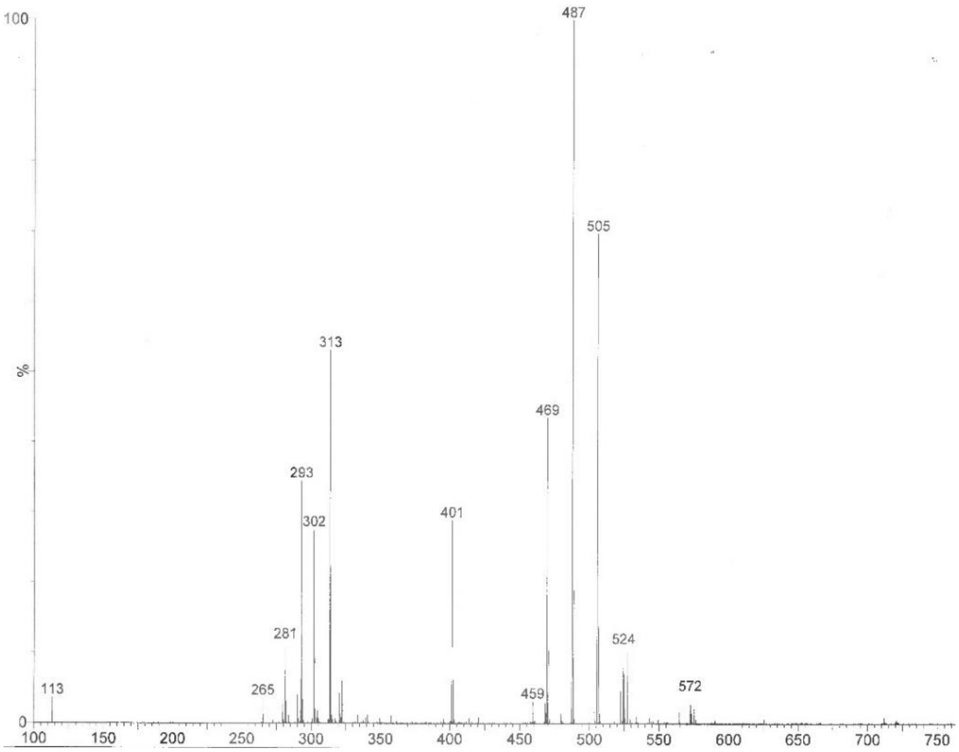

Supplementary Figure 12. Mass spectrum of MAEDA monomer.

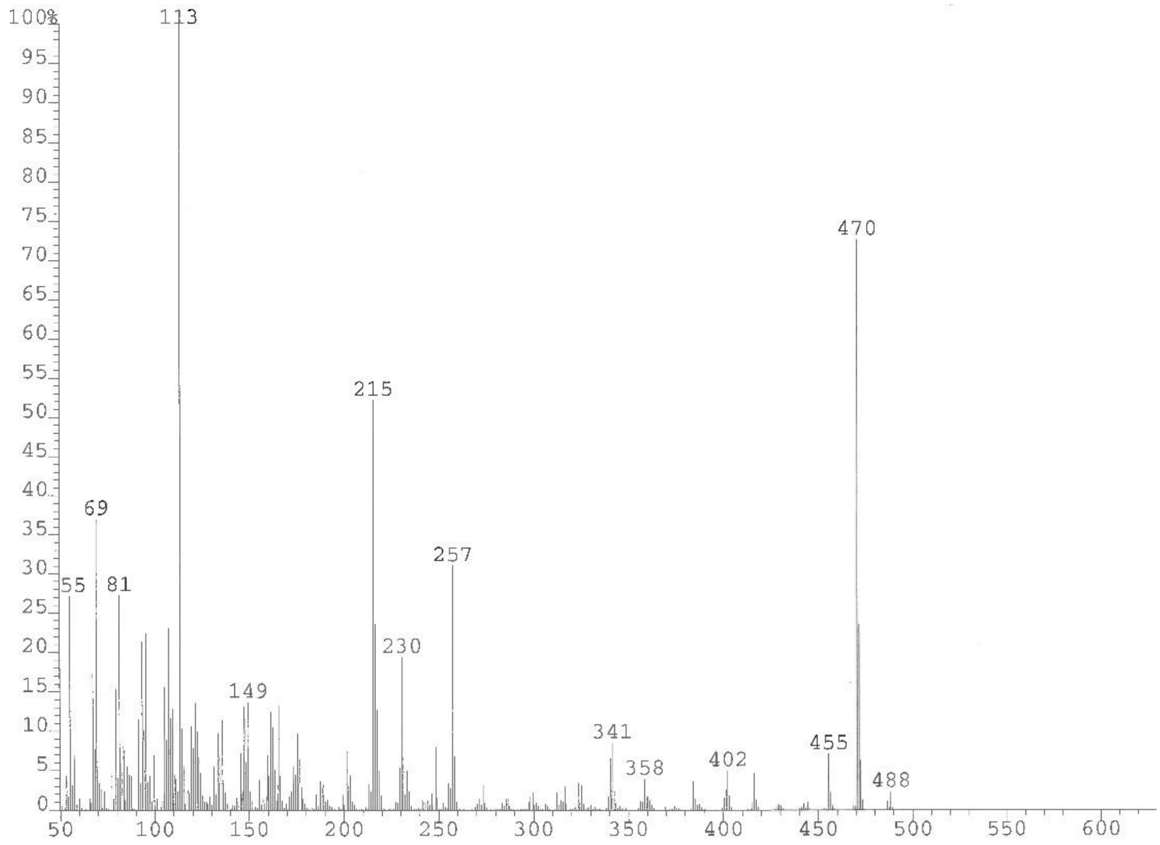

Supplementary Figure 13. Mass spectrum of MAELA monomer.

Supplementary Table

Supplementary Table 1. Molecular weight and dispersity of bile acid-derived polymers.

Polymer Symbol	M_n (g mol^{-1}) (GPC) Before post-polymerization modification	$Đ$	M_n (g mol^{-1}) (GPC) After post-polymerization modification	$Đ$
CA_19k_5	19,000	1.10	23,000	1.10
DCA_19k_5	19,000	1.11	22,000	1.14
LCA_19k_5	20,000	1.12	21,000	1.12
CA_10k_5	10,000	1.07	13,000	1.15
CA_19k_3	19,000	1.10	21,000	1.13
CA_19k_1	19,000	1.10	19,000	1.10
CA_25k_5	25,000	1.16	38,000	1.23
CA_32k_5	32,000	1.26	45,000	1.28

Supplementary References

1. Ganewatta MS*, et al.* Bio-inspired resin acid-derived materials as anti-bacterial resistance agents with unexpected activities. *Chem Sci* **5**, 2011-2016 (2014).

2. Qiao Y, Yang C, Coady DJ, Ong ZY, Hedrick JL, Yang Y-Y. Highly dynamic biodegradable micelles capable of lysing Gram-positive and Gram-negative bacterial membrane. *Biomaterials* **33**, 1146-1153 (2012).

3. Lam SJ*, et al.* Combating multidrug-resistant Gram-negative bacteria with structurally nanoengineered antimicrobial peptide polymers. *Nat Microbiol* **1**, 16162 (2016).
